# A method for probabilistic mapping between protein structure and function taxonomies through cross training

**DOI:** 10.1186/1472-6807-8-40

**Published:** 2008-10-03

**Authors:** Kshitiz Gupta, Vivek Sehgal, Andre Levchenko

**Affiliations:** 1 The Whitaker Institute for Biomedical Engineering, Johns Hopkins School of Medicine, Baltimore, MD, USA; 2 Department of Computer Science & Engineering, Indian Institute of Technology, Bombay, Mumbai, India; 3 Department of Computer Science, University of Maryland, College ParkCollege Park, MD, USA; 4 Yahoo! Inc., 701 First Avenue, Sunnyvale, CA, USA

## Abstract

**Background:**

Prediction of function of proteins on the basis of structure and vice versa is a partially solved problem, largely in the domain of biophysics and biochemistry. This underlies the need of computational and bioinformatics approach to solve the problem. Large and organized latent knowledge on protein classification exists in the form of independently created protein classification databases. By creating probabilistic maps between classes of structural classification databases (e.g. SCOP [1]) and classes of functional classification databases (e.g. PROSITE [2]), structure and function of proteins could be probabilistically related.

**Results:**

We demonstrate that PROSITE and SCOP have significant semantic overlap, in spite of independent classification schemes. By training classifiers of SCOP using classes of PROSITE as attributes and vice versa, accuracy of Support Vector Machine classifiers for both SCOP and PROSITE was improved. Novel attributes, 2-D elastic profiles and Blocks were used to improve time complexity and accuracy. Many relationships were extracted between classes of SCOP and PROSITE using decision trees.

**Conclusion:**

We demonstrate that presented approach can discover new probabilistic relationships between classes of different taxonomies and render a more accurate classification. Extensive mappings between existing protein classification databases can be created to link the large amount of organized data. Probabilistic maps were created between classes of SCOP and PROSITE allowing predictions of structure using function, and vice versa. In our experiments, we also found that functions are indeed more strongly related to structure than are structure to functions.

## Background

Function and 3D structure of the proteins are said to be related to each other [3]. However, prediction of function on the basis of structure and vice versa still remains a partially solved problem, and is largely in the domain of biophysics and biochemistry [4]. This underlines the need for computational and bioinformatics methods to establish relationships between functions and structures of proteins. Previous attempts have been largely limited to examining a single protein and predicting structure and function based on its size, charge, sequence, and other physical attributes [5-7]. Further, content knowledge of protein classification has also been used to predict structure and function using data mining techniques [8-10]. Large protein classification schemes (e.g. SCOP [1], CATH [11], PROSITE [2], Pfam [12]) are available in public domain in the form of protein classification databases. Arguably, this latent knowledge has not been sufficiently used to relate structure and function by establishing relationships between the various schemes. Various classifiers are built using data mining techniques using the above latent knowledge to designate a given protein to a structural or a functional class. We propose that probabilistic linking of these classification databases could be used to establish relation between function and structure of proteins. In addition, individual classes in widely used protein databases could be linked together to further consolidate the large amount of classification data on proteins.

Growing proteomics data have motivated the design of many schemes to classify proteins. Proteins can be classified according to a variety of classification schemes based on features like proteins domains [13], structure [1,11], phylogeny [14], ligand binding sites [15], subcellular localization [16,17] etc. In addition to the schemes based on biologically defined features, many schemes are based on abstractions that are expected to correlate with biological families (e.g. functional signatures [2,18], sequence motifs [19]). Intuitively, in all these schemes there would exist a *semantic overlap*. In other words, different schemes of classification may not be completely independent of each other, and relationships may exist between classes of different taxonomies. For instance, proteins sharing a certain motif may also belong to a common phylogenetic family.

Protein classification is performed by either manual annotation [1], or automatic classification based on defined feature sets [2,9,20,21]. Common classifiers like Support Vector Machines (SVM) [22-25], Bayesian classifiers [20,26] and others have been used to classify proteins using attributes like primary sequences, size, localization [16] etc. Existing methods for protein classification include profiles for protein families [27], pairwise sequence alignment [28], consensus patterns using motifs [19] and hidden Markov models [29,30]. Though discriminative classifiers (e.g. SVM) in general have higher accuracy, generative models (e.g. hidden Markov models) have been preferred over discriminative models since variable length of protein sequence data renders it difficult to use discriminative classifiers [31]. SVMs have been shown to outperform other basic classifiers like Naive Bayes [32] in accuracy. Further, no assumption of the domain knowledge is required to train SVM [23]. If the domain knowledge is correctly known, it can be incorporated to improve accuracy like in Fischer-SVMs [24] and SVM-mismatch kernels [33], or even in non-linear SVM [34].

With protein databases being populated at an astonishing pace, it has become essential to consolidate the knowledge latent in the existing and emerging databases. Presently these relationships can only be established by manual annotations using static accession numbers. These numbers (e.g. SwissProt accession number [35]) link protein entries in different classification databases, without linking the classes in the databases [36]. This underlines the need for computational methodologies to identify relationships between various schemes, even if it is probabilistic. In this paper we present a novel method to establish these relationships between classes of taxonomies in a probabilistic manner between two widely used and independent classification schemes: PROSITE and SCOP. We demonstrate that this method could be effectively used to establish probabilistic relations between functional and structural classes of proteins. PROSITE [2] and SCOP [1,37] are well annotated taxonomies based on functional motif and structure of proteins respectively. The relationships between PROSITE and SCOP are not understood and completed and have never been explored systemically before. There has been no attempt to *cross learn *using existing two classifications and identify relationships between the two. It would be, therefore, instructive to look at relationships between the two classifying schemes and assess if there is a semantic overlap between the two independent classification systems.

We have modified previously reported cross-training algorithm by introducing a hierarchy based approach [38] to apply to biological databases. The method involves simultaneous training of two different sets of classification schemes using a classifier iteratively, till semantic overlaps cannot be utilized for further enhancement in accuracy. We used the existing taxonomies of PROSITE and SCOP and mutually trained them using hierarchical cross training. SVM was used as a classifier employing a variety of attributes including a few that have been designed for this special purpose. The motivation is to classify protein into a known functional taxonomy, PROSITE, when the placement in a known structural taxonomy, SCOP, is known.

## Results and discussion

**Figure 1:**
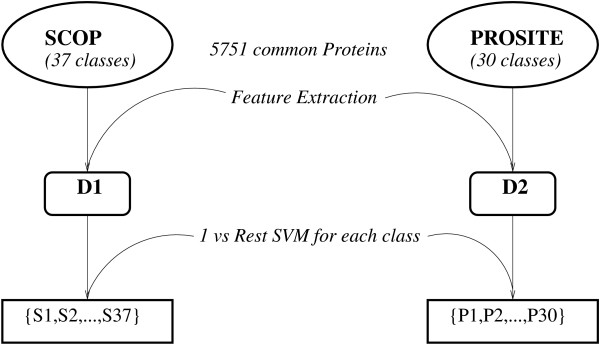
**SVM training of SCOP and PROSITE**. 5751 protein instances common to both PROSITE and SCOP were taken to train the respective SVMs. 30 most populated classes in PROSITE and 37 most populated classes in SCOP were used. Randomly half of 5751 proteins were used to train the SVM classifier for PROSITE and other half for SCOP. Blocks, elastic 2-D profile, molecular mass, size, percentage of helices, *β *chain were used as orthogonal features for 1 vs rest SVM training for each class.

Partial taxonomies from SCOP and PROSITE were used for hierarchical cross training using the features and procedures described in METHODS section. SwissProt [35] database was taken for feature extraction. 5751 proteins common to both PROSITE and SCOP were used to train the respective SVMs. 30 most populated classes in PROSITE, each class being a *domain *[39], and 37 most populated classes in SCOP, each class being a *superfamily *[40], were used in experiments. The most populated class in PROSITE contained 102 proteins while the least populated amongst the used 30 classes contained 24 proteins. Out of the 5751 proteins considered, randomly half were used for training of the SVM classifier for PROSITE and the other half for SCOP (Figure 1).

In our experimentations, *linear *support vector machine (SVM) was used as the classifier. SVMs have been shown to outperform other basic classifiers like Naive Bayes [32] in accuracy, are discriminative classifiers and require no assumption regarding domain knowledge. The chief advantage of using SVM is that it is easily scalable and inclusion of new dimensions does not affect the accuracy of the classifier. This property is very useful when large numbers of features are used for training, as in our experimentations. Further, hierarchical cross training requires introduction of new dimensions in the classifier (see METHODS), easily achievable in SVM. For all the above reasons, SVM was chosen as the classifier.

The training of SVM is a bit expensive, of the order *n*^*k *^(typically, 1.8 ≤ k ≤ 2.1) but the testing is still linear and the high accuracy advantage overrides the disadvantage of higher training time [22,23].

### Metrics

Performance evaluation for most of the functional classes using structural classes as features resulted in high *recall *(Equation 2) and *precision *(Equation 3) values. These two quantities were unified into a single quantity called *F*-measure (Equation 1) for analysis (Table 1, 2, 3). F-measure is the weighted harmonic mean between recall and precision, both being evenly weighted. For a given class *A*, F-measure was defined as follows:

**Table 1:** Comparison between F measure while using Blocks and k-length subsequences

**Class**	**F (subsequences)**	**F (Blocks)**
Small proteins	.78	.73
Globin like	.71	.684
NAD P binding Rossmann fold domains	.54	.537

Comparison of F-measure using k-length overlapping subsequences of length (k) equal to 4 and blocks as features. In the case of using blocks as features, F-measure was found to be only slightly lower for all the classes, while the size of feature set was many times smaller.

**Table 2:** Performance evaluation for some functional classes

Class Name	FP Rate	Precision	Recall	**F-measure**
Immunoglobulins and MHC protein	0.002	0.765	0.619	**0.684**
Ig-like domain profile	0.005	0.547	0.343	**0.422**
Cytochome c family, heme-binding site signature	0	0.988	0.908	**0.946**
Globins family	0.002	0.792	0.623	**0.697**
Pyridine nucleotide-disulphide oxidoreductases c-I	0.001	0.704	0.559	**0.623**
Serine protease trypsin family	0.001	0.918	0.789	**0.848**
ATP dependent helicases signatures	0.002	0.52	0.419	**0.464**
Phospholipase A2 active site signature	0	1	0.862	**0.926**
Nuclear hormones receptors DNA-binding	0.003	0.667	0.561	**0.61**

Performance evaluation while trying to classify functional classes using structural classes as the features. The high F-measure values (Equation 1) indicate that the function is strongly dependent on the structure that the protein has. F-measure was calculated as the harmonic mean between Recall and Precision. Class-name refers to *domain *in PROSITE database. Highly significant F-measure values are shown in bold.

**Table 3:** Performance evaluation for some structural classes

Class-Id	FP Rate	Precision	Recall	**F-measure**
*α *&*β*.NADP-bind Rossmann fold	0.008	0.25	0.259	0.254
Immunoglobulin *β *sandwich	0.018	0.296	0.282	0.289
*α*.globin like	0.005	0.649	0.61	**0.629**
Small proteins	0.063	0.245	0.216	0.213
Peptides	0.017	0.242	0.18	0.207

Performance evaluation while trying to classify structural classes using functional classes as the features. The low F-measure values indicate that though structure is dependent on function of the protein, the present features are incapable in distinguishing them completely. F-measure was calculated as the harmonic mean between Recall and Precision. Presented table lists classes which showed significant F-measure values. Class-name refers to *superfamily *in SCOP database. Highly significant F-measure values are shown in bold.

(1) $$F-measure=\frac{2*\text{Recall}*\text{Precision}}{\text{Recall}+\text{Precision}}$$

(2) $$Recall_{A}=\frac{\text{proteins correctly found in A}}{\text{total proteins in A}}$$

(3) $$Precision_{A}=\frac{\text{proteins predicted correctly in A}}{\text{total proteins predicted in A}}$$

### Employing novel features for training resulted in reduced time complexity without compromising performance

Blocks and 2D elastic profile were used as novel features to train the SVM classifiers for SCOP and PROSITE. As detailed in the METHODS section, blocks were preferred over k-length subsequences as features for training classifiers (see METHODS). Using the value of *k *as 4 in overlapping k-length subsequences resulted in more than 100000 unique features. To reduce the number of above features, Principle Component Analysis (PCA) [41] was used to discover principle components that define most of the data variability. Application of PCA resulted in reduction of unique features to 1000. k-length subsequences are subsequences of optimal size that are able to capture most information represented in the sequence of the protein. In addition, the k-length subsequence as a feature does not give additional weight to subsequences that occur commonly and are conserved amongst polypeptides. Hence, we employed a novel feature for classifiers called blocks [42]. Blocks are multiple aligned ungapped segments corresponding to the most highly conserved regions of proteins. In blocks [42] database, for each query protein a 'distance' is calculated against the corresponding most conserved homologous block. Nearly 10,000 unique blocks were generated for PROSITE dataset, and PCA application reduced it to 100. For nearly all classes, F-measure (Equation 1) obtained using blocks as features were similar or slightly lower than the case when k-length subsequence were used as features (Table 1). Statistically speaking, a classifier using blocks as features will have lower chances of "over-fitting" as the features are fewer in number. Due to all the above advantages without significant reduction in the F-measure, blocks were used as features instead of k-length subsequence in our experimentations.

### Hierarchical cross training indicates semantic overlap between SCOP and PROSITE

**Figure 2:**
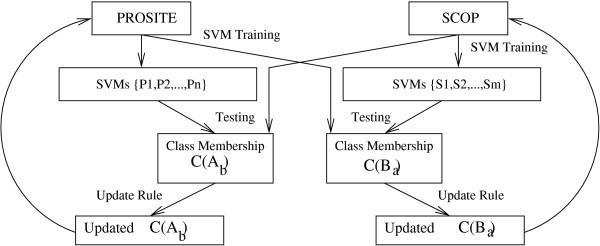
**Cross training flow**. Datasets are generated by cross training, where the taxonomy A (or B) had features as classes from taxonomy B (or A) respectively. In effect, classifier of PROSITE is trained using classes of SCOP as features and vice versa. SVM classifiers were created for both PROSITE and SCOP (Figure 1). Classes of PROSITE were used as features for SCOP and protein feature vector was updated. Similarly, classes of SCOP were used as features for classifier of PROSITE and protein feature vector was updated. Cross training was iterated till further gain in accuracy stops.

Supervised cross training as a concept was introduced in Chakrabarti *et al *[38]. If we have two taxonomies A and B with strong semantic overlap, then information from A can be used to train B and vice-versa (Figure 2). The approach not only helps in improving accuracy but can also be used to learn relationships between classes belonging to different taxonomies. To establish a baseline, we trained taxonomies of SCOP and PROSITE using linear SVM classifiers with the set of features described earlier. Hierarchical cross training of the taxonomies of SCOP and PROSITE resulted in an average increase of 5.2% in F-measure for classes in the two taxonomies. This improvement in accuracy obtained by cross training PROSITE and SCOP classifiers demonstrates that a semantic overlap exists between the classes of the two taxonomies. Further, it establishes that using information across taxonomies improves learning, particularly in the case of functional and structural classification schemes. It was found that a cross-trained SVM outperforms standard SVM and is specially effective in the case when baseline accuracy levels are low. This was found to be true for the structural classifiers which have low accuracy levels. Results are summarized in Additional File 1.

F-measure obtained for classes in functional classifier using structural classes as features were high, and exhibited low false positive rates (Table 2). For a few classes like *Cytochrome c family, heme-binding site signature *and *Phospholipase A2 active site signature*, the F-measure was close to 0.95 on a 1.00 point scale with 0 false positives indicating high confidence in establishing relationships from SCOP to PROSITE. This indicates that the position of a given protein in a functional class in PROSITE is strongly dependent on the position of the protein in a SCOP class.

On the other hand, F-measure for classes in structural classifier using functional classes as features were found to be low (Table 3). Most of the classes, with the exception of *All alpha protein.Globin-like *superfamily, showed F-measure less than 0.30 on a 1.00 scale. Similarly, the false positives were higher than encountered in the cross training product of PROSITE signature classes. This suggests that either the structural properties are not highly dependent on the function of the proteins, or the present features are incapable in distinguishing them completely. Intuitively, it seems correct, that it is with a greater confidence that the function of the protein is predicted given the structure, while it is not easy to predict the structure of a protein given the function.

### Decision Trees for SCOP and PROSITE relate two taxonomies in a probabilistic manner

Hierarchical cross training produced a complex mapping (many to many) between classes of SCOP and PROSITE. Decision trees were constructed to provide human visualization between structural and functional classes of proteins, and to extract probabilistic relationships between them (see METHODS). If membership of a given protein in PROSITE (or SCOP) is known, it can be used to find its class in SCOP (or PROSITE). A probabilistic weighted *score *was generated based on the decision tree.

Decision trees were obtained for both PROSITE (DT-PROSITE) and SCOP (DT-SCOP). DT-PROSITE had SCOP classes as features and DT-SCOP had PROSITE classes as features. DT-PROSITE showed low false positive rates (Table 2) and high F-measure lending credibility to the decision tree and the rules (of the form *classes*-*in*-*SCOP → class*-*in*-*PROSITE*) to be generated from it.

Simplification of the rules has generated direct relationships from classes in SCOP to PROSITE and vice versa with a probabilistic weighted *score*. A few significant relationships are shown in Table 4. Rules like *All alpha proteins. Globin-like *→ *Globins family profile *showed a high score (Table 4) suggesting strong relationships between the two classes. Significantly, a reverse relationship was also found with a high score from PROSITE to SCOP. It is noteworthy that the colloquial names for both classes also implied that they were related. A similar case was found in the rule *All beta proteins.Cupredoxin-like *→ *Type-1 copper blue proteins signature *with a high score. *All alpha proteins.Globin-like *superfamily in SCOP also suggested a strong relationship to the *Legume lectin signature *in PROSITE, a rule also found in DT-SCOP with a high score. Similarly, rules were obtained relating classes SCOP to classes in PROSITE like *α *&*β proteins. Thioredoxin fold → Pyridine nucleotide-disulphide oxidoreductases class-I active site with a score of 0.097*, *All β Proteins.Nucleoplasmin-like VP viral coat & capsid proteins → DEAD & DEAH box families ATP-dependent helicases signatures *with score of 0.14.

**Table 4:** Prediction rules between classes in SCOP and PROSITE obtained by cross-training

**Rules: Structural-properties (SCOP class) ⇒ Functional-properties (PROSITE class)**
All *α *Proteins.Globin like → Globinsfamily profile **0.323**
All *α *Proteins.Globin like → Legume lectins signatures **0.079**
All *β *Proteins.Cupredoxin like → Type-1 copper blue proteins signature **0.200**
All *β *proteins.Galactose-binding domain-like →C-type lectin domain signature & profile **0.182**
All *β *Proteins.Nucleoplasmin-like VP viral coat & capsid→ DEAD & DEAH box families ATP-dependent helicases signatures **0.14**
*α *&*β *proteins.NADP- binding Rossmann-fold domains → C-type lectin domain signature & profile **0.103**
All *β *proteins.Double-stranded *β*-helix → EF-hand calcium-binding domain **0.086**
All *α *Proteins.EF Hand like → EF-hand calcium-binding domain **0.090**
All *β *Proteins.Immunoglobulin-like *β *sandwich → Immunoglobulins & MHC proteins **0.034**
All *β *Proteins.Immunoglobulin-like *β *sandwich → Ig-like domain profile **0.064**
*α *&*β *Proteins.*α β*-Hydrolases → EF-hand calcium-binding domain **0.086**
*α *&*β *proteins.Ferredoxin-like → 4Fe-4S ferredoxins iron sulfur binding region signature **0.024**
*α *&*β*proteins. P-loop containing nucleoside triphosphatehydrolases → Heavy-metal-associated domain signature & profile **0.037**
*α *&*β *proteins.Thioredoxin fold → Pyridine nucleotide-disulphide oxidoreductases class-I active site **0.097**
All *α *proteins.Four-helical up-&-down bundle → Cytochrome c family heme-binding site signature **0.094**
Membrane & cell surface proteins and peptides → C-type lectin domain signature & profile **0.092**
All *β *proteins.Concanavalin A-like lectins glucanases → C-type lectin domain signature & profile **0.084**
All *α *proteins.DNA RNA-binding 3-helical bundle → TNFR NGFR family cysteine-rich region signature & profile **0.062**
Small proteins → Serine proteases trypsin family signatures & profile **0.010**
Small proteins → Ig-like domain profile **0.047**

**Rules: Functional-properties (PROSITE class) **⇒ **Structural-properties (SCOP class)**

Globins family profile → All *α *proteins.Globin-like **0.369**
Serine proteases trypsin family signatures & profile → Small proteins **0.722**
Protein kinases signatures & profile → Small proteins **0.662**
Legume lectins signatures → All *α *proteins.Globin-like **0.060**
Cytochrome c family heme-binding site signature → All *β *proteins.Barrel-sandwich hybrid **0.029**

Selected prediction rules found as a by-product of cross-training SCOP and PROSITE. Top panel shows rules probabilistically predicting the PROSITE domain/signature given the SCOP superfamily, while the bottom panel shows rules predicting SCOP superfamily given the PROSITE domains. DOT ('.') links parent class to child class in SCOP hierarchy. The bold value following each rule is the probabilistic weighted score found using cross training.

In addition, we found that two PROSITE classes, *Zinc finger RING-type signature and profile *and *Zinc finger C2H2-type domain signature and profile*, occurred together in most of the rules. This leads us to project that these signatures are highly related signatures and are commonly found in the proteins in which they occur. It is noteworthy that these classes occur commonly, and presence of these signatures together in proteins may have potential biological significance.

A few inferences can be drawn from the generated rules listed in Table 4. Structural classes, as properties, have a higher bearing on the functional classification for proteins than vice versa. Few classes in SCOP and PROSITE were obtained that were related in the form *class-in-SCOP *→ *class-in-PROSITE *AND *class-in-PROSITE *→ *class-in-SCOP *simultaneously with high probabilistic scores. Further, a few classes like *C-type lectin domain signature and profile *and *Zinc finger RING-type signature and profile *in PROSITE occur more commonly than others as the rhs (right hand side) of rules with high scores. Statistically it means that proteins having a structural property (left hand side of the rule) were found to contain features commonly found in proteins belonging to the said classes in PROSITE.

## Conclusion

In this paper, we describe a methodology to establish structured relationships between two independent protein taxonomies using hierarchical cross training of classifiers for each taxonomy. Previous studies have described classifiers developed for various protein taxonomies using a variety of features [33]. However, no attempt to cross train two taxonomies using the classes of one as a feature to train the classifier for the other taxonomy has been made so far. We demonstrated that strong semantic overlaps exist between SCOP and PROSITE, in spite of the independent classification schemes. It should be noted that SCOP is a manually annotated taxonomy, while in PROSITE annotation is automated. Hierarchical cross training allows the knowledge of label assignment in one taxonomy to be used in another taxonomy and establish relationships between the two. This, therefore, is a novel attempt to link two widely used protein classification databases and find probabilistic relationships between the classes of either. SCOP and PROSITE have different taxonomical structures and different ways of static classification of proteins, having evolved entirely independently of each other. Since SCOP is a structural classification and PROSITE is essentially a functional classification, our method also provides a non commutative map between functional and structural classes of proteins, rendering it the first datamining effort in this direction.

Blocks and 2D elastic profile are novel features used to train the decision tree and are more informative than k-length subsequences. Blocks resulted in reduced feature set, time complexity of SVM without compromising performance. This may be because most of the k-length subsequences were not adding extra information and only a few were actually participating in classification. Most of the other classifiers use the whole sequence or overlapping standard sized subsequences as features, rendering the feature set extremely large. The number of features used for training in our method is therefore small making the method fast yet effective. Since the accuracy of the classifier for functional classes using structural classes as features is very high, we believe that these features could also be used as standard features for protein classification mechanisms. However, better and more informative features might be necessary to train structural classifier using functional classes as features. In addition, non linear SVMs (polynomial [22], or radial basis function [34]) may be used to improve the accuracy of classifiers. Though no such exercise has been performed using cross training on protein taxonomies, but we believe that other structural and functional protein databases can be cross trained using our method to generate more informative rules.

F-measure of certain classes is lower than other classes since proteins belonging to one PROSITE class may belong to multiple SCOP superfamilies. The extent of this mismatch is difficult to assess since large number of proteins are not known in their 3D structures. Similarly, many proteins in the SCOP database do not have conserved sequence motifs identifiable in the PROSITE database, and thus can not be assigned to a PROSITE class. A probable reason is that PROSITE only collects well established sequence motifs with significant population in the sequence databases. In such cases, the method prediction here would provide lower confidence for prediction of relationships. Hence, we neglected rules below a certain threshold probabilistic score.

In addition, it must be remembered that the cross training described here is aimed at showing the efficacy of the methodology, and hence is performed on partial taxonomies (5751 proteins) of SCOP and PROSITE. Many more rules can be generated if the method is applied to the complete taxonomies. It is unlikely that the presented rules would change appreciably since most of the remaining classes in SCOP and PROSITE are sparsely populated. Further, it must be noted that the method is essentially a data mining effort, and reflects any inherent bias of the taxonomies on which it is conducted. Such biases could occur due to the biased research in favor of proteins that are already discovered, are more relevant to human pathology, or biased evolution in proteomics in favor of certain classes of proteins. In addition, a repetition of hierarchical cross training for PROSITE and CATH [11], another hierarchical structural classification database may generate more informed relationships between functions and structures of proteins. It would be instructive to find out semantic overlaps and generate probabilistic maps between classes of taxonomies that are based on function, but different schemes, eg. PROSITE and Pfam [12]. We would like to envisage the bigger goal to generate extensive "probabilistic linkage maps" between various prominent protein classification databases which can be updated in time. Typically proteins are linked only through accession ids of databases and no static link can be developed between classes in different taxonomies [36]. Therefore, a probabilistic linkage between classes of proteins in different databases would be a significant step forward to link the whole of proteomic data [36,43,44].

## Methods

Relationships between classes were discovered as a *by-product *of cross training. The approach can be broadly divided into two parts. The first part deals with feature extraction and representation of a protein to train the classifiers for both PROSITE and SCOP. The second part involves hierarchical cross training and extraction of relationships between classes of PROSITE and SCOP.

### Feature Sets

A variety of features are typically used in training a classifier. These choices are mostly empirical and intuitive and making these choices is a non trivial problem with significant bearing on the accuracy of classification [22]. We have used novel features detailed below to train our classifiers.

### Subsequences

Previous attempts have included fixed and variable length subsequences as feature sets [45]. Consecutive and overlapping subsequences of length *k *are chosen as features. However, k being small would result in lower accuracy, while a large *k *would lead to over-fitting. Therefore, a locally-optimal value of *k *was chosen to maximize the accuracy of classifier and enhance its statistical significance.

S*ubroutine to find optimal k:*

Dataset with the primary sequence = *DP*

mean-ss = 0

*k *= 0

while (mean-accuracy increase) ≥ 0 and (ss ≥ mean-ss) do

   *k *= *k *+ 1

   Create *D *from *DP *with sequence features of length k

   for i = 1 to 10

      (TR [i], TE [i]) = Split dataset D in *train *and *test *sets

      Train a classifier(SVM) CL using training data TR [i]

      accuracy [i] = test classifier CL on testing data TE [i]

   end for

   mean-accuracy = mean of accuracy [i] for *i *= 1 to 10

   Calculate ss for this set using the t-test.

   mean-ss = (mean-ss*(k-1) + ss)/k

end while

The value of statistical significance *ss *was defined as

(4) $$\begin{array}{cc} ss=\frac{{\left(N\right)}^{1/2}}{\text{Standard error}}, & number\;of\;iterations\text{ N}=10 \end{array}$$

Optimal *k *was found to be 4 on PROSITE dataset. For a given protein *p*_*i *_the count of a k-length subsequence *f *was defined as

(5) $$Count(f,p_{i})=\sum_{j=1}^{j<L/K}Occ(f,j,p_{i})*Active(j,p_{i})$$

where *L *is the length of the complete protein sequence

(6) $$Active(j,p_{i})=\{\begin{array}{ll} Constant\text{ c }(=10), & j\text{ is an}\\ & \text{active site};\\ 1, & \text{otherwise} \end{array}$$

(7) $$Occ(f,j,p_{i})=\{\begin{array}{ll} 1 & \text{if }f\text{ overlaps position}\\ & {\text{k}}^{*}j\text{ in }p_{i};\\ 0, & \text{otherwise} \end{array}$$

*Count *is the approximate number of occurrences of the feature *f *in a protein *p*_*i*_. To introduce added weightage to the active sites in the protein, the occurrence, *Occ*, was counted multiple times (*c *times). SwissProt [35] entries were used to determine the active site. The value of *c *was taken as 10 in our experimentations.

### Blocks

Blocks were defined as features and count was calculated as

(8) *Count *= *Block length*/(1 + *Block distance*)

where *distance *is the dissimilarity index

with the most conserved

corresponding block

This definition ensures that more weightage is given to larger blocks, which are assumed to preserve more biological information. Further, weightage is inversely proportional to the block distance (dissimilarity index) with the most conserved block [42].

### 2-D elastic profile

Previous attempts to use secondary structure as features for protein classification have been mostly limited to utilization secondary structure content [46,47], or localized secondary structure [48]. No previous attempt in our knowledge has been made to use the global secondary profile of the protein as a feature. One of the reasons is that proteins have variable lengths which makes the comparison difficult. This problem was solved by introducing a notion of elastic secondary structure. The secondary structure profile was extracted from SwissProt and was linearly scaled to a length of 100 resulting in an 'elastic' profile through stretching or compressing. Here the number 100 was chosen just for convenience. Intuitively, it also behaved like a global feature, as it was not only influenced by changes in the locality but also by additions or deletions at other locations in the protein.

Formally, for a protein *p *of size *L*, a secondary structure array was defined as

(9) $$\begin{array}{c} 2^{0}\text{struct}[i]=\{\begin{array}{ll} 1 & \text{if }strand\text{ at location }i\\ 2 & \text{if }turn\text{ at location }i\\ 3 & \text{if }helix\text{ at location }i\\ NAN & otherwise \end{array}\\ \text{for }i\text{ from }1\text{ to }L. \end{array}$$

Then using this array the 2-D elastic feature was defined as

(10) $$\begin{array}{c} 2\text{-D elastic}[i]=2^{0}\text{ struct}\left[\left\lfloor L/100*i\right\rfloor \right]\\ \text{for }i\text{ from }1\text{ to }100 \end{array}$$

### Other Features

Molecular mass, size, percentage of helices, beta strands in the whole protein etc. were other features used for classification. One column/dimension was maintained for each feature. Value of each feature was either equal to the absolute value (like mass in case of molecular-mass) or it was binary (1, if the feature was present; 0 otherwise). Equal interval binning was used for many features (e.g. percentage of helices, beta strands etc.) to allow generalization.

### Final representation

A protein was represented as a vector of all the above features. This representation is based on an assumption that features are orthogonal to each other. This assumption was made for the sake of time efficiency and to reduce the complexity of algorithm.

### Hierarchical Cross-training

Hierarchical cross training on SVM involves introduction of new artificial dimensions/features to distinguish between the otherwise indistinguishable instances using normal feature sets. So if A-classes are a good predictors of B-classes, classification accuracy of proteins in B may be improved by allocating for each protein in B a set of new columns/features, one for each A-class (Figure 2). Hence, the altered protein ${p^{\prime }}_{i}$ is represented as:

(11) $${p^{\prime }}_{i}=\left(\begin{array}{c} p_{i}\\ Cm_{i}^{T} \end{array}\right)$$

Here, $Cm_{i}^{T}$ refers to the enhanced feature set for each protein in B obtained from classes in A. While adding new dimensions to the protein feature-vector, an assumption is made that the kernel space remains orthogonal. Specifically, the new set of dimensions *Cm*_*i *_are also orthogonal to all other features. Since protein classes are taken from a hierarchy, this assumption is not entirely true. This concern was addressed by modifying the algorithm and adapting it for hierarchical biological taxonomies.

Firstly, one-vs-rest SVMs are trained for each class. For training a non-leaf class the positive data used is present within the descendant leaf nodes, while the rest of the data is taken as negative examples. While dealing with a *hierarchy *during cross training, the basic idea used was that a protein that belongs to a child class also belongs to the corresponding parent class. To be more specific, let *p *be a protein, *c *any class and *Ansc*_*c *_be set of all classes ancestor to class *c*. Two cases arise:

1. *Rule*1: *p ***has a high probability to belong to class ***c*: Then *p *has a high probability to belong to the ancestor classes *Ansc*_*c *_too.

2. *Rule*2: *p ***has a low probability to belong to class ***c*: In this particular case, nothing can be said about *p*'s relation with the ancestor classes *Ansc*_*c*_.

C*ross Train Algorithm:*

Train SVMs for A-classes (*C*_*A*_) using

   proteins from dataset-A (*D*_*A*_).

Train SVMs for B-classes (*C*_*B*_)

   using proteins from dataset-B (*D*_*B*_).

Each protein *p*_*i *_in *D*_*B *_is classified

   using *C*_*A *_and the corresponding

   class-membership vector (*Cm*_*i *_= $\left(cm_{i}^{1},...,cm_{i}^{n}\right)$)

   (class-membership represents the probability of an

   instance belonging to various classes in a

   taxonomy) is calculated.

   Here $cm_{i}^{j}$ is the SVM score obtained

   by 'testing' protein *p*_*i *_with SVM

   for the *j*^*th *^class.

**Update-Protein: **Using the class-membership *Cm*_*i*_

   for every protein in B, the protein features

   are updated using protein update rule.

Similarly repeat the above steps for proteins in A.

Retrain *C*_*A *_using the modified proteins from *D*_*A*_.

Retrain *C*_*B *_using the modified proteins from *D*_*B*_.

Return to step 3 if there is increase in

   classification accuracy of *C*_*A *_and *C*_*B*_.

The above information is incorporated in the protein update rule. Further, it needs to be established when does a given protein belongs to a particular class *c *with "high probability". One simple way of estimation is by calculating the class-membership vector *Cm*_*p *_for any given protein *p *by testing it with the SVM-classifier for each class. The class with the maximum positive value in *Cm*_*p *_is defined as the only class to which the protein *p *belongs to with "high probability". This method is, however, naive and would miss the correct class in case more than two classes have high and close positive values. Also, during experimentations it was found that in many instances the entire *Cm*_*p *_vector is negative and hence no single positive value exists. A softer version was therefore developed which can replace the cross training update rule where *Cm*_*p *_was re-scaled and then the above two rules were used to update the membership values of the ancestor classes.

S*ubroutine to update Protein Vector:*

*I/P *: protein *p*, *O/P *: updated protein

Calculate the class-membership vector *Cm*_*p*_.

**Rescaling step: **Find maximum class-membership

   value *val*_*max*_. Add (1 - *val*_*max*_)

   to each element in the vector. This step will

   ensure a positive value for at least one class.

**Identifying high probability classes: **Find all

   classes *C*_*p *_for which class-membership

   value is positive.

**for **every class *c *∈ *C*_*p *_{

   Let the class-membership value of *c *is *val*_*c*_.

   Find the ancestor classes *Ansc*_*c*_.

**Updating ancestor classes: **Increase

   class-membership for each class in *Ansc*_*c *_by *val*_*c*_.}

end Subroutine

### Extracting relationship using the decision tree

The decision tree [49] algorithm induces a series of comparison in form of a binary tree, where each non-leaf node is expressed as a comparison of a feature *f*_*i *_(classes from taxonomy A) value with a constant value. The comparison decides whether to go to either the left or right subtree. The leaf-nodes are classes to which the instant can belong to (classes from taxonomy B). Hence, if we know the corresponding membership in one taxonomy for a protein, it can be used to find its class in the other taxonomy. The advantage of this approach is that the protein is not required to belong to only a single class and the user can input the strength for each class. A probabilistic weighted score is generated based on the decision tree. We employed the decision tree algorithm to find out the probability of proteins belonging to a class in SCOP to belong to a given class in PROSITE, and vice versa. This created a probability map from SCOP to PROSITE, and vice versa, linking all the classes in either taxonomy to each other with a probabilistic weight. Since PROSITE is a functional classification scheme and SCOP is a structural classification scheme, by corollary, the above probabilistic map can be construed as a probabilistic map between functional and structural properties of proteins.

Subroutine to create decision tree:

*A *&*B *are taxonomies.

*D*_*A *_= dataset for *A *after full cross-training with *B*.

Calculate class-membership vector *Cm*_*i*_∀*p*_*i *_∈ *D*_*A*_

   using classes in *B*.

Represent every protein *p*_*i *_in A using *Cm*_*i*_.

   Call it $D_{A}^{d}t$.

Train a decision tree *DT*_*A *_using this dataset $D_{A}^{d}t$.

Repeat the above steps for B to get decision tree *DT*_*B*_.

Each path in *DT*_*A *_is a rule

   *classes*-*in*-*B *→ *class*-*in*-*A*.

Each path in *DT*_*B *_is a rule

   *classes*-*in*-*A *→ *class*-*in*-*B*.

end Subroutine

## Authors' contributions

KG and VS devised the idea, implemented the project, conducted experiments and drew conclusions. AL supervised the project and drafting of the manuscript.

## Supplementary Material

Additional file 1 **Baseline test for hierarchical cross training**. Linear SVM based classifiers were used to train SCOP and PROSITE without using classes of the other taxonomy as features. In a subsequent test, hierarchical cross training algorithm was used to train the classifiers using the same feature sets (detailed in Methods section). An average increase of 5:2% in F-measure was obtained after employing hierarchical cross training. The table lists the old (without hierarchical cross training) and new (with hierarchical cross training) F-measure obtained for a few illustrative classes. Click here for file
