# Supplementary material for: A method for probabilistic mapping between protein structure and function taxonomies through cross training
Source: BMC Struct Biol. 2008 Oct 3;8:40. doi: 10.1186/1472-6807-8-40 (PMC2573881; doi:10.1186/1472-6807-8-40)
Supplement: Additional file 1 — Baseline test for hierarchical cross training. Linear SVM based classifiers were used to train SCOP and PROSITE without using classes of the other taxonomy as features. In a subsequent test, hierarchical cross training algorithm was used to train the classifiers using the same feature sets (detailed in Methods section). An average increase of 5:2% in F-measure was obtained after employing hierarchical cross training. The table lists the old (without hierarchical cross training) and new (with hierarchical cross training) F-measure obtained for a few illustrative classes. [file 1472-6807-8-40-S1.doc]

**Supplementary Table 1: Baseline test for hierarchical cross training**

F-old | F-new | Class

------------------------

0.684 0.647 _Immunoglobulins_and_major_histocompatibility_complex_proteins_

0.233 0.315 _EF-hand_calcium-binding_domain_

0.048 0.024 _Eukaryotic_thiol__cysteine__proteases_active_sites_

0.102 0.163 _Neutral_zinc_metallopeptidases__zinc-binding_region_signature_

0.422 0.474 _Ig-like_domain_profile_

0.946 0.876 _Cytochrome_c_family_heme-binding_site_signature_

0.000 0.039 _Platelet derived_growth_factor__PDGF__family_signature_and_profile_

0.047 0.152 _Small_cytokines__intercrine_chemokine__signatures_

0.059 0.212 _Cytosolic_fatty-acid_binding_proteins_signature_

0 0.143 _Lipocalin_signature_

0 0.215 _C-type_lectin_domain_signature_and_profile_

0.697 0.857 _Globins_family_profile_

0 0 Zinc_finger_RING-type_signature_and_profile_

0.042 0 Tyrosine_specific_protein_phosphatases_signature_and_profiles_

0 0.118 _4Fe-4S_ferredoxins__iron-sulfur_binding_region_signature_

0.407 0.333 _Type-1_copper__blue__proteins_signature_

0.623 0.531 Pyridine_nucleotide-disulphide_oxidoreductases_class-I_active_site_

0 0.045 2Fe-2S_ferredoxins__iron-sulfur_binding_region_signature_

0.848 0.841 Serine_proteases__trypsin_family__signatures_and_profile_

0 0.033 TNFR_NGFR_family_cysteine-rich_region_signature_and_profile_

0.095 0.061 Eukaryotic_and_viral_aspartyl_proteases_signature_and_profile_

0.202 0.275 Protein_kinases_signatures_and_profile_

0 0.128 Zinc_finger_C2H2-type_domain_signature_and_profile_

0 0.032 Heavy-metal-associated_domain_signature_and_profile_

0.149 0.341 Src_homology_3__SH3__domain_profile_

0.111 0.163 _Ubiquitin_domain_signature_and_profile_

0.464 0.656 _DEAD_and_DEAH_box_families_ATP-dependent_helicases_signatures_

0.113 0.243 _Legume_lectins_signatures_

0.926 0.873 _Phospholipase_A2_active_sites_signatures_

0.61 0.617 _Nuclear_hormones_receptors_DNA-binding_region_signature_
